# *Prunus amygdalus var. amara* seed extract enhances the antileishmanial activity of miltefosine

**DOI:** 10.1186/s12906-025-04958-z

**Published:** 2025-07-16

**Authors:** Sajjadul Kadir Akand, Areeba Rahman, Rahat Ali, Mohammad Husain, Mohd Danish, Mohammad Rashid Khan, Nemat Ali, Mohd Faiz Akram, Abdur Rub

**Affiliations:** 1 Department of Biotechnology, Jamia Millia Islamia (A Central University), New Delhi, 110025 India; 2 Department of Dermatology, University of Alabama, 1720 2nd Ave South, Birmingham, AL35294 USA; 3 Department of Pharmacology and Toxicology, College of Pharmacy, King Saud University, P.O. Box 2457, Riyadh, 11451 Saudi Arabia; 4 Faculty of Dentistry, Department of Pharmacology, Jamia Millia Islamia (A Central University), New Delhi, India 110025

**Keywords:** Leishmaniasis, Prunus amygdalus var. amara seed, Leishmania, Miltefosine, Apoptosis, IC_50_

## Abstract

**Background:**

Leishmaniasis, an infectious disease transmitted via sand flies is caused by the protozoan parasite of *Leishmania* *spp*. The treatment of this disease is quite challenging due to the high cost, resistance, and toxicity of conventional drugs. Various research studies have demonstrated that plant based drug possess least toxicity, anti-inflammatory and anti-oxidant properties. Here, evaluation of anti-leishmanial activity of methanolic *Prunus amygdalus var. amara *seed extract was conducted and found that it inhibited *L. donovani* proliferation and cause apoptosis. Moreover, its combinations with miltefosine enhanced antileishmanial effects. GC–MS analysis confirmed the presence of various phytochemicals in the extract that contributed pharmacological efficacy. These findings highlighted the potential of herbal products as a valuable source of new treatments for leishmaniasis.

**Methods:**

The antileishmanial effect was determined by promastigote and amastigote assays. Parasite load was evaluated by staining *L. donovani-*infected macrophages with modified Giemsa stain. Cytotoxicity of seed extract was estimated by MTT (3-(4, 5- dimethylthiazol-2-yl)-2, 5-diphenyl tetrazolium bromide) assay. In addition, Pro-apoptotic events were inferred using RT-PCR and qRT-PCR. Further characterization of phytoconstituents was evaluated by gas chromatography and mass spectrometry.

**Results:**

The extract promoted a dose-dependent reduction in growth of promastigotes (IC50 = 43.12 ± 3.03 μg/ml) and amastigotes (IC50 = 49.65 ± 3.34 μg/ml). Further, extract in combination with miltefosine showed enhanced antileishmanial activity against both forms of the *L. donovani*, promastigotes (IC50 = 4.547 ± 1.2 μg/ml) as well as amastigotes (IC50 = 19.54 ± 2.4 μg/ml). Early-stage apoptotic events were also observed in promastigote forms by determining the increased expression of LdMetacaspase and PARP1. The cytotoxic potential on THP-1 differentiated macrophages was assessed and indicated insignificant cytotoxicity of different doses of the extract (CC50 = 799.19 ± 134.59 μg/ml) and in combination with miltefosine (CC50 = 384.16 ± 177.47 μg/ml). Furthermore, the presence of phytocompounds like chaulmoogric acid and hydnocarpic acid was described, for the first time, in *Prunus amygdalus* var. *amara* seed extract.

**Conclusion:**

The findings indicated that EPA plays a significant role in combating leishmaniasis and holds promise as a potential treatment for this disease. Moreover, when combined with miltefosine, EPA demonstrated increased effectiveness against leishmaniasis. Therefore, the combination of EPA and miltefosine presents a more promising outlook as a potential therapy for leishmaniasis.

**Supplementary Information:**

The online version contains supplementary material available at 10.1186/s12906-025-04958-z.

## Background

Leishmaniasis is a group of vector-borne diseases caused by the intracellular protozoan parasite *Leishmania* and transmitted by female sandflies mainly *Lutzomyia Franca* and *Phlebotomus Rondani* with a diverse range of clinical manifestations [1, 2]. Visceral leishmaniasis (VL) or kala-azar, is a lethal, chronic neglected tropical disease (NTD) caused by *Leishmania donovani* complex, *L. donovani* in the Indian subcontinent and East Africa, and *L. infantum* in Latin America, Europe, and North Africa [3]. VL is a visceral infection of the reticuloendothelial system that becomes fatal if left untreated [4]. VL is ranked second in the mortality rate among NTD [5, 6]. Currently, there is no vaccine available for leishmaniasis in humans. According to WHO, leishmaniasis is causing 700,000 to one million new cases annually. Further, its transmission is documented in 98 nations and three territories with a preliminary estimate of 20,000 to 40,000 deaths annually [7]. In addition, Leishmaniasis primarily affects individuals in underprivileged areas of the world, i.e., developing and underdeveloped countries, yet the chemotherapy for the disease is expensive, ranging from 30 to 1500 dollars for medications alone [8]. The drug used currently in the Indian subcontinent, sodium antimony gluconate showed no response in more than 64% of the patients due to the development of resistance against the parasites [9]. Alternate drugs, miltefosine, and amphotericin B, and their lipid formulations have several limitations because of high toxicity, cost, and unavailability, which limit their use. The currently available Leishmaniasis treatment and therapy are cumbersomebecause it has several drawbacks, including the cost factor, drug availability in remote areas, inherent species-specific drug susceptibility differences, and due to relative drug efficacy between geographical areas [10]. Furthermore, the lack of vaccination for the condition exacerbates the situation. Various new strategies should be implemented to combat the disease and overcome the above challenges. Since ancient times, Plants have been known for various medicinal properties and are the least toxic as compared to synthetic drugs [11]. Numerous plant extracts have been shown to be effective against a range of infectious and non-infectious disorders. *Aerva lanata* extract, for instance, has been discovered in recent investigations to have potential antifungal action against *Candida albicans* and *Aspergillus niger* [12]. Similar to this, many plants, like *Viscum continuum* and *Eucalyptus globulus*, have antibacterial characteristics [13, 14]. Additionally, plant extracts can be used to treat viral infections [15, 16]. Furthermore, it has been claimed that plant extracts such as *Menyanthes trifoliata L., and Angelica sylvestris* may possess anti-cancerous qualities [17] Owing to the above arguments we searched for a plant that may exhibit the better anti-leishmanial activity with the least cytotoxicity and further possible mechanism of action can be elucidated. Hence, online screening of hundreds of plants was conducted based on various scientific findings demonstrating their antimicrobial properties, pharmacological constituents, and their availability.*Prunus amygdalus var. amara*belongs to the family Rosaceae (rose family) and its seed has the same physical characteristics as sweet almonds except shorter and broader in size and bitter taste [18]. A considerable number of flavonoids are present in the Prunus amygdalus var. amara seed extract [EPA]. Behzad Moradi et.al. stated that aqueous extract, Methanolic extract, and ethanolic EPA have varied antimicrobial effects. Of these methanolic extract is more effective on Bacillus subtilis and Staphylococcus aureus [19]. Hamid Abtahi et.al marked in their study that the highest synergistic effect of EPA is detected in methanol and aqueous extracts [20, 21]. Furthermore, EPA has shown considerable antifungal properties. In addition, the oil of *Prunus amygdalus var. amara* seed is said to be the best cure for renal pain, urinary bladder stones, and dysuria [21]. Here, the study investigates the antileishmanial activity of EPA alone and in combination with miltefosine through various in vitro assays.

## Materials and methods

### Reagents and chemicals

RPMI 1640, M199 Media, Fetal bovine serum (FBS), Phosphate Buffered Saline (PBS) and Penicillin–streptomycin antibiotic were procured from Gibco, Thermo Fisher Scientific, Erie N2 UK. Trizol, Sodium bicarbonate, and HEPES from Sigma Aldrich, USA; cDNA synthesis kit, 2X power up SYBR green master mix from Thermo Fisher Scientific, Erie N2 UK, Phorbol 12-myristate 13-acetate (PMA) was purchased from Peprotech, USA. Gene-specific primers were purchased from Integrated DNA Technologies Pte. Ltd. paraformaldehyde was obtained from Sigma-Aldrich, Saint Louis, MO. Molecular Biology Grade DEPC treated, Nuclease and Protease free water was from HiMedia. DNA ladder was procured from simplybiologics. Miltefosine, 3-[4,5-dimethylthiazol-2-yl]−2,5 diphenyl tetrazolium bromide (MTT) assay reagents, and cell culture-grade dimethyl sulfoxide (DMSO) and other solvents for plant extract were from Merck & Co., Inc., Kenilworth, NJ. Modified Giemsa stains were obtained from Merck Life Science. Inc Propidium. 2 × green master mix procured from Promega. Power up Sybr green master mix procured from Thermo.

### Sample collection and identification

The Plant part was collected from a local Bangalore market and transported to Jamia Millia Islamia. Botanical identification was done and verified by Dr. Sunita Garg, Chief Scientist and Head, Herbarium dept. (NIScPR/RHMD/Consult/2022/4119–20) NISCAIR, New Delhi, India.

### Preparation of EPA

An electric grinder was used to grind the dried kernel of seed. The powdered substance was then dissolved in 240 ml of methanol in a 500 ml conical flask and shaken continuously for 72 h at room temperature. Using Whatman filter paper, the solution was filtered, and the filtrate was then concentrated using a rotatory evaporator under vaccum at 35^0^ C.The seed extract was stored at 20^0^C. [22] A 100 mg/ml stock solution was made by combining 100 mg of EPA with 1 ml of culture-grade DMSO.

### Promastigote phase parasites and THP-1 cell line culture and maintenance

The infective strain of *Leishmania donovani* (MHOM/IN/83/AG83) was maintained in M199 media at pH 7.4 supplemented with 25 mM HEPES, 10% heat-inactivated FBS, and 1% penicillin–streptomycin antibiotic cocktail. The log phase parasite culture was passaged at regular intervals of 72 to 96 h with the inoculum density of 2 × 10^6^ parasites per ml at 22^0^C. THP-1 human monocytic cell line was maintained in RPMI-1640 media supplemented with 10% FBS and 1% penicillin–streptomycin antibiotic in a humidified atmosphere in a CO^2^ incubator at 5% CO^2^ and 37 ^0^C temperature. THP-1 monocytic cells were stimulated by 20 ng/ml of phorbol myristate acetate (PMA) for differentiation to macrophages.

### In vitro anti-promastigote activity evaluation of EPA alone and in combination with miltefosine

L. donovani logarithmic-phase-promastigotes (2 × 10^6^ parasites/ml) were incubated in a 6-well plate in M199 media with different concentrations of extract(45 μg/ml, 135 μg/ml,250 μg/ml, 405 μg/ml) and in combination with 2 μg/ml miltefosinefor 72 h. Parasites were fixed in 1% paraformaldehyde, and viable parasites were counted through the Neubauer chamber with a coverslip. Untreated parasites served as the negative control. Further, 2 μg/ml miltefosine was taken as the positive control. 50% inhibitory concentration (IC50) was determined by plotting the graph between doses versus viability using graphpad prism software [23].

### Cytotoxicity evaluation of EPA alone and in combination with miltefosine through MTT assay

For cell cytotoxicity assay, 2 × 10^5^ THP-1 cells/ml were seeded in each well of a 96-well plate and stimulated with 20 ng/ml of PMA for 24 h to differentiate into macrophages. After 24 h of incubation, the differentiated adherent cells were washed with blank RPMI 1640 media and were treated with different doses of seed extract alone (45 μg/ml, 135 μg/ml,250 μg/ml) and in combination with 2 μg/ml miltefosine for 24 h. Cells were further incubated with 20 µl of 5 mg/ml of MTT reagent (3-(4, 5- dimethylthiazol-2-yl)- 2, 5-diphenyl tetrazolium bromide) for 4 h at 37 °C in a CO_2_ incubator followed by dissolving the resulting formazan crystal in 100 µl of DMSO. The amount of formazan produced represents the relative number of viable cells. The absorbance was read at OD 570 nm on an ELISA reader (Thermo Scientific Varioskan). The cell viability graph was plotted between viability versus concentration and the 50% cytotoxic concentration (CC50) was measured by a dose–response curve using graphpad prism. The selectivity index (SI) was determined by the CC50/IC50 values ratio (Table 1).

**Table 1:** The gas chromatography-mass spectrometry (GC/MS) analysis of methanolic EPA

S. No	Compound Name	Area %	Retention Index	Molecular weight	Molecular Formula	Nature of Compound
1	Methyl Palmitate	0.50	1878	270	C_17_H_34_O_2_	Esters
2	3-Allyal-1-cyclooctene	0.04	0	150	C_11_H_18_	Alkene
3	Dibutyl phthalate	0.16	2037	278	C_16_H_22_O_4_	Benzoic acid esters
4	Methyl 11-(2-cyclopenten-1-yl)undecanoate	7.72	1904	266	C_17_H_30_O_2_	Fatty acid
5	**Hydnocarpic acid**	**68.17**	1993	252	C_16_H_28_O_2_	Fatty acid
6	5,7-Dodecadiene	0.10	1230	166	C_12_H_22_	Alkene
7	Methyl 7-octadecenoate	0.39	2085	296	C_19_H_36_O_2_	Ester
8	Methyl gorlate	1.49	2110	292	C_19_H_32_O_2_	Ester
9	Methyl hydnocarpate	2.55	1904	266	C_17_H_30_O_2_	Fatty acid
10	Gorlic Acid	3.25	0	278	C_18_H_30_O_2_	Glyceride
11	**Chaulmoogric acid**	**15.02**	0	280	C_18_H_32_O_2_	Fatty acid
12	Docosane	0.49	0	310	C_22_H_46_	Alkane
13	Euracamide	0.15	2625	337	C_22_H_43_NO	Fatty acid Amide

### In vitro anti-amastigote activity evaluation of EPA alone and combination with miltefosine

For conducting an amastigote assay, 0.5 × 10^6^ cells/well were seeded in a 6-well plate with coverslips, and PMA (20 ng/ml) was added in wells for differentiation to macrophages. The plate was incubated at 37^0^C overnight in a CO_2_ incubator followed by removing the undifferentiated cells and adding fresh media. Further, metacyclic promastigotes, the infective form of the parasite, were added in each well in the ratio of 1:10 and were kept at 37 ^0^C in a CO_2_ incubator for 12 h. The non-internalized parasites were washed out with 1X PBS buffer and the infected macrophages were treated with different concentrations of the extract alone (45 μg/ml, 135 μg/ml,250 μg/ml) and in combination with 2 μg/ml miltefosine and kept for the next 48 h. The coverslips were washed with 1X PBS, fixed with chilled methanol for 30 min, and stained by using a modified Giemsa stain for amastigote microscopic evaluation. About 100 macrophages were counted from different focuses to evaluate the effect of the treatment on the macrophagic parasite burden.

### RNA isolation and cDNA synthesis

6 × 10^6^ log phase promastigotes/well were seeded in a 6-well plate and treated with different doses of EPA alone and in combination with miltefosine for 24 h.The sample was homogenized in 500 µL of TRIzol reagent and incubated at room temperature for 5 min. Chloroform (100 µL) was added, followed by centrifugation at 12,000 rpm at 4^0^C for 15 min to separate the phases, and the aqueous phase containing RNA was collected. RNA was precipitated with 250 µL of chilled isopropanol by centrifugation at 12,000 rpm at 4^0^C for 10 min, washed with 75% chilled ethanol, and resuspended in 20 µL RNase-free water. cDNA was synthesized from 1 μg of RNA using a verso cDNA synthesis kit as per the manufacturer’s protocol.

### RT-PCR and qRT-PCR

Primers specific to L.donovani pro-apoptotic genes were designed through the NCBI primer designing tool and ordered. The primer pairs LdMetacaspase forward, 5′ AAA CGG GTC GAC ATT AAT GC 3′ and LdMetacaspase reverse 5′ CGA GCA TGA GGA AAA GAT CA3′, LdPARP1 forward, 5′ TGCCGGAAGGCGGCTCATTC 3′ and LdPARP1 reverse, 5′ CGCAGTGCGTTGCGCATACC 3. were checked for its optimal Tm through gradient PCR. Gene expression analysis of the genes was done through RT-PCR and electrophoresed the PCR product in 1.5% agarose gel containing EtBr. qRT-PCR of the genes was performed through Sybr green dye and gene expression was analysed through Quantstudio 6 software.

### GC–MS examination of the EPA

The secondary metabolites that may be in charge of EPA antileishmanial activity were found using GC–MS analysis. Seeds were powdered, and extracted in methanol. It was then examined using the prescribed methods on a Shimadzu QP2010; GCMS-QP2010 SE: SHIMADZU (Shimadzu Corporation) equipped with a DB-5MS column at Jawaharlal University, New Delhi, India. The sample's mass spectra were generated in the 70 eV electron impact ionization mode, and the phytochemicals were determined by comparing the recorded mass spectrum to the reference libraries WILEY8.LIB and NIST14.LIB was supplied with the GC–MS system's software.

### Statistical analysis

All the experiments were performed in triplicate and the results represented are the mean of the triplicate with SE. Statistical analysis was performed using Graph pad prism 8.0 software and a P-value of less than 0.05 was considered significant. The statistical significance was calculated using one-way ANOVA followed by student t-test.

## Result

### EPA enhances the antileishmanial effect of miltefosine on *L. donovani* promastigotes

**Fig. 1:**
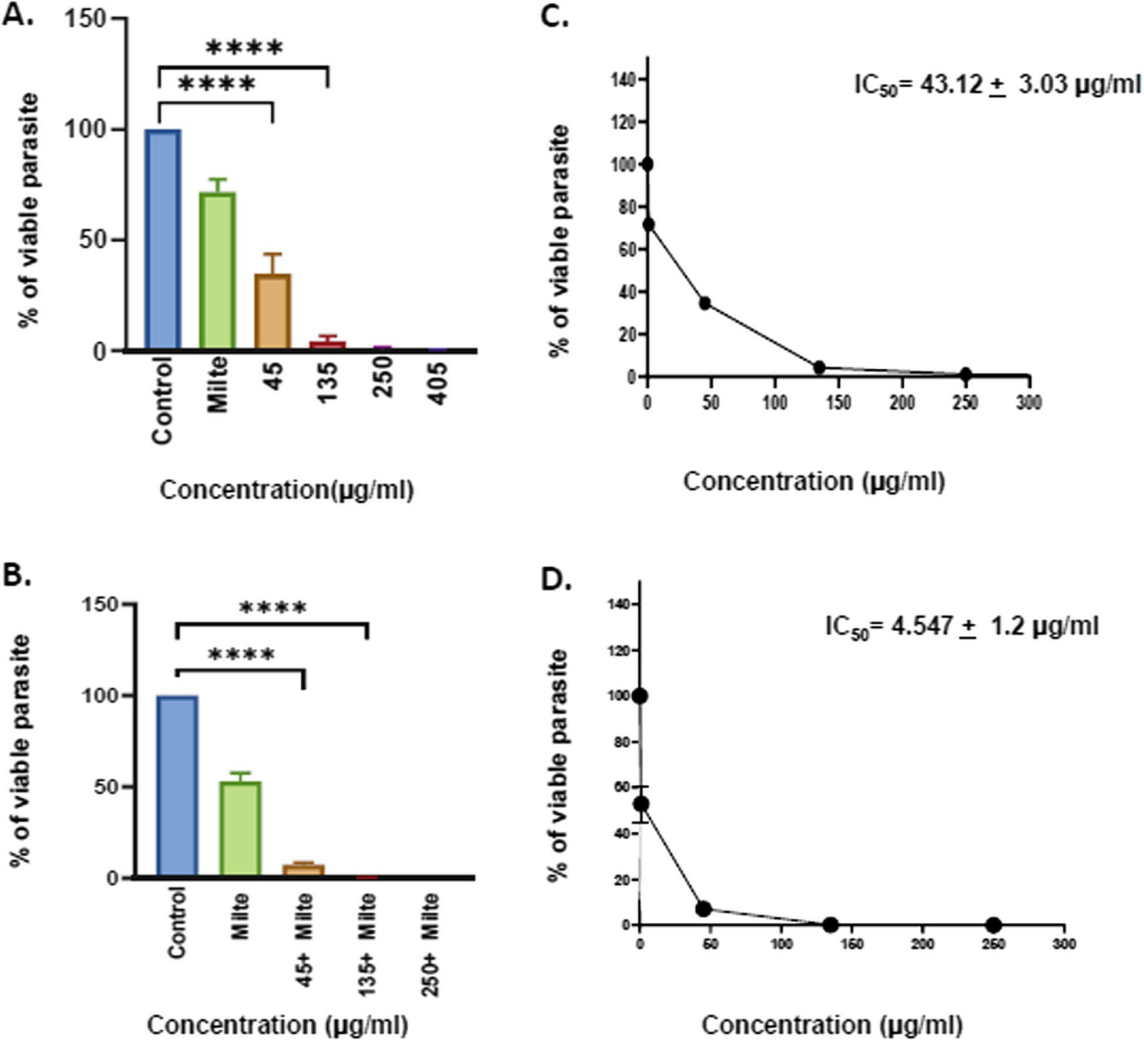
Antileishmanial activity of EPA by promastigote assay.** A** 2 X 10^6^ stationary phase *L. donovani* promastigotes were treated with different doses of methanolic EPA. **B ***L. donovani* promastigotes were treated with different doses of EPA in combination with Miltefosine. **C **IC50 value was calculated in case of parasite treated with methanolic EPA at different doses. **D **IC50 value was calculated in case of parasite incubated with methanolic EPA in combination with miltefosine at different doses.Each set is statistically significant compared to control. Untreated promastigotes were taken as control while miltefosine was taken as positive control

The growth inhibitory effects of the methanolic EPA against exponentially growing *L. donovani* promastigotes were accessed. The effect of EPA in combination with miltefosine on *L. donovani* promastigotes was also studied. EPA treatment reduced the number of promastigotes in a dose-dependent manner.It was observed that at doses 45 µg/ml, 135 µg/ml 250 µg/ml, and 405 µg/ml of EPA, the number of promastigotes were highly reduced at 72 h of treatment as compared to the control (Fig. 1A). Miltefosine, a standard antileishmanial drug was taken as a positive control. Further, EPA was used in combination with 2 µg/ml of miltefosine for better antileishmanial efficacy. It was observed that the combinatorial effect of 45 µg/ml EPA with 2 µg/ml miltefosine significantly reduces the number of promastigotes compared to the control while 135 µg/ml EPA + 2 µg/ml miltefosine and 250 µg/ml + 2 µg/ml miltefosine reduces the number of promastigotes to ~ 0% as compared to control (Fig. 1B). The IC_50_ value of EPA on promastigotes was calculated as 43.12 ± 3.03 µg/ml (Fig. 1C). Similarly IC_50_ value of EPA in combination with miltefosine was calculated and found to be 4.547 ± 1.2 µg/ml (Fig. 1D).

### Combined doses of EPA with miltefosine showed insignificant cytotoxicity on THP-1 derived macrophages

**Fig. 2:**
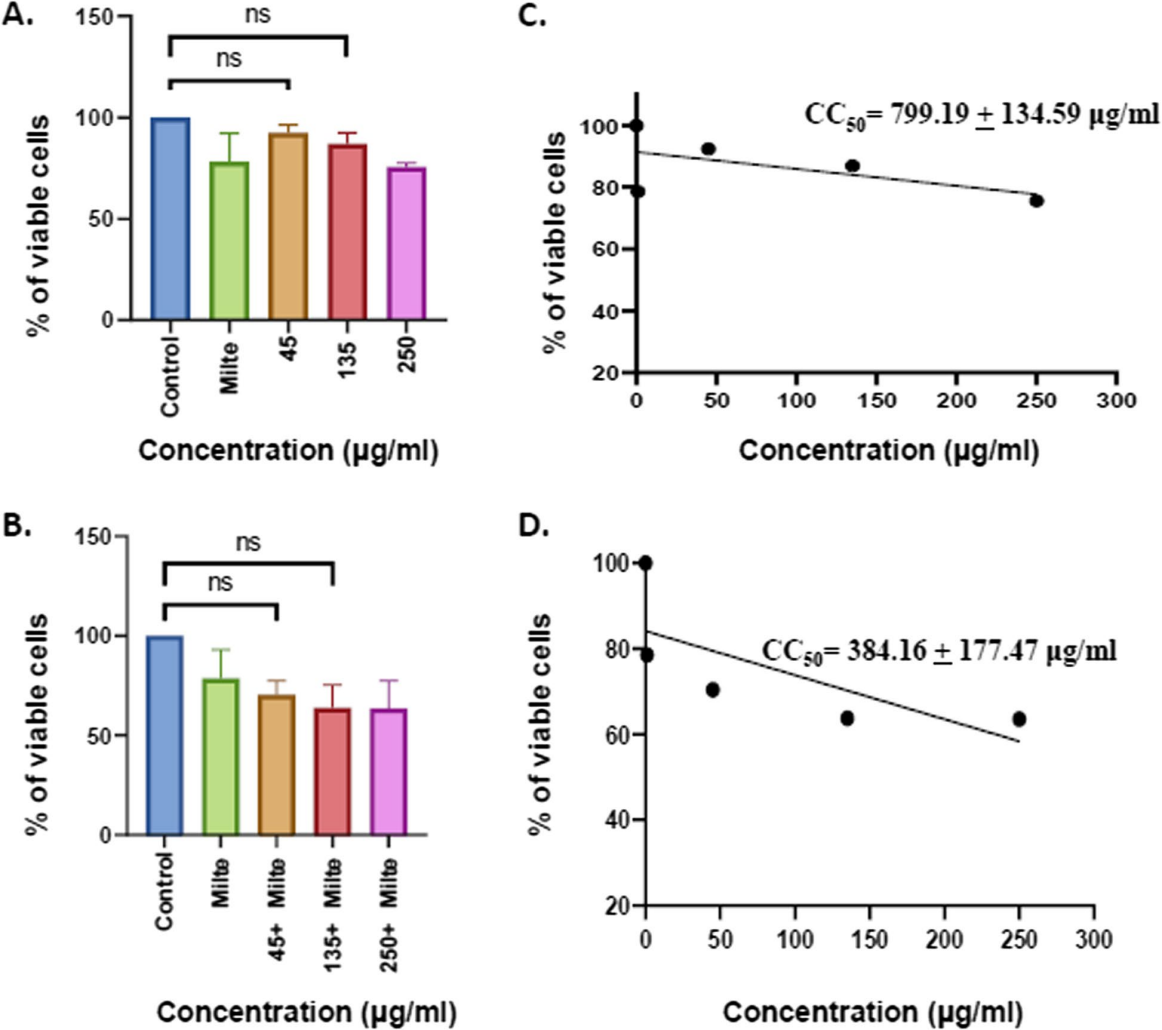
Cytotoxicity of EPA on THP-1 differentiated macrophages by MTT assay. **A** THP-1-differentiated macrophages were treated with different concentrations of EPA, **B** THP-1-differentiated macrophages were treated with different concentrations of EPA in combination with constant dose of 2 µg/ml miltefosine, **C** CC50 value was calculated in case of THP-1 differentiated macrophage treated with methanolic EPA at different doses, **D** CC50 value was calculated in case of THP-1 differentiated macrophage incubated with methanolic EPA in combination with miltefosine at different doses. Untreated THP-1differentiated macrophages were taken as control while miltefosine was taken as positive control

MTT assay was performed to evaluate the cell cytotoxicity (CC_50_) of methanolic EPA alone and in combination with the standard drug miltefosine on THP-1 differentiated macrophages. It was observed that more than 50% of cells are viable in all the doses of EPA alone as well as in combination (Fig. 2A & B). The CC_50_ value of EPA alone and in combination was calculated to be 799.19 ± 134.59 μg/ml and 384.16 ± 177.47 μg/ml (Fig. 2 C & D) respectively.

### EPA along with miltefosine significantly reduced intramacrophagic amastigotes

**Fig. 3:**
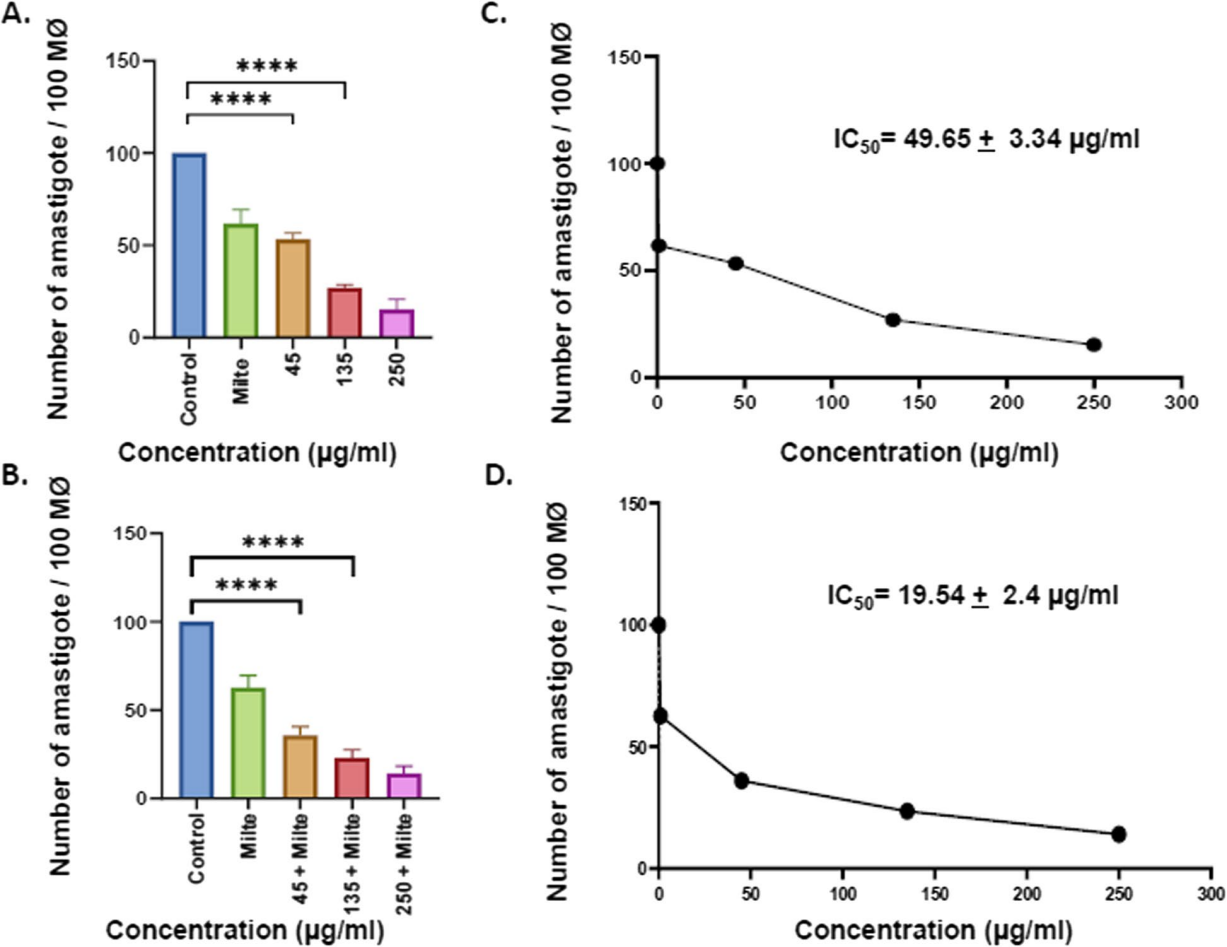
EPA in combination with miltefosin decreased the parasite load in macrophage.** A** THP-1-differentiated macrophages were parasitized in a 1:10 ratio with stationary phase promastigotes and then treated with different concentrations of EPA. **B **Intramacrophagic parasites were treated with increased dose of EPA (45µg/ml to 250 µg/ml) along with a constant dose of miltefosine (2 µg/ml) in *L. donovan,. ***C** IC50 value was calculated in case of intramacophagic parasites treated with methanolic EPA at different doses, **D** IC50 value was calculated in case of intramacrophagic parasites incubated with methanolic EPA in combination with miltefosine at different doses.Percent reduction in the parasite load was determined as described in the method. ****P < 0.0001, value was statistically significant as compare to control. Untreated promastigotes were taken as control while miltefosine was taken as positive control.Percent reduction in the parasite load was determined as described in the method

**Fig. 4:**
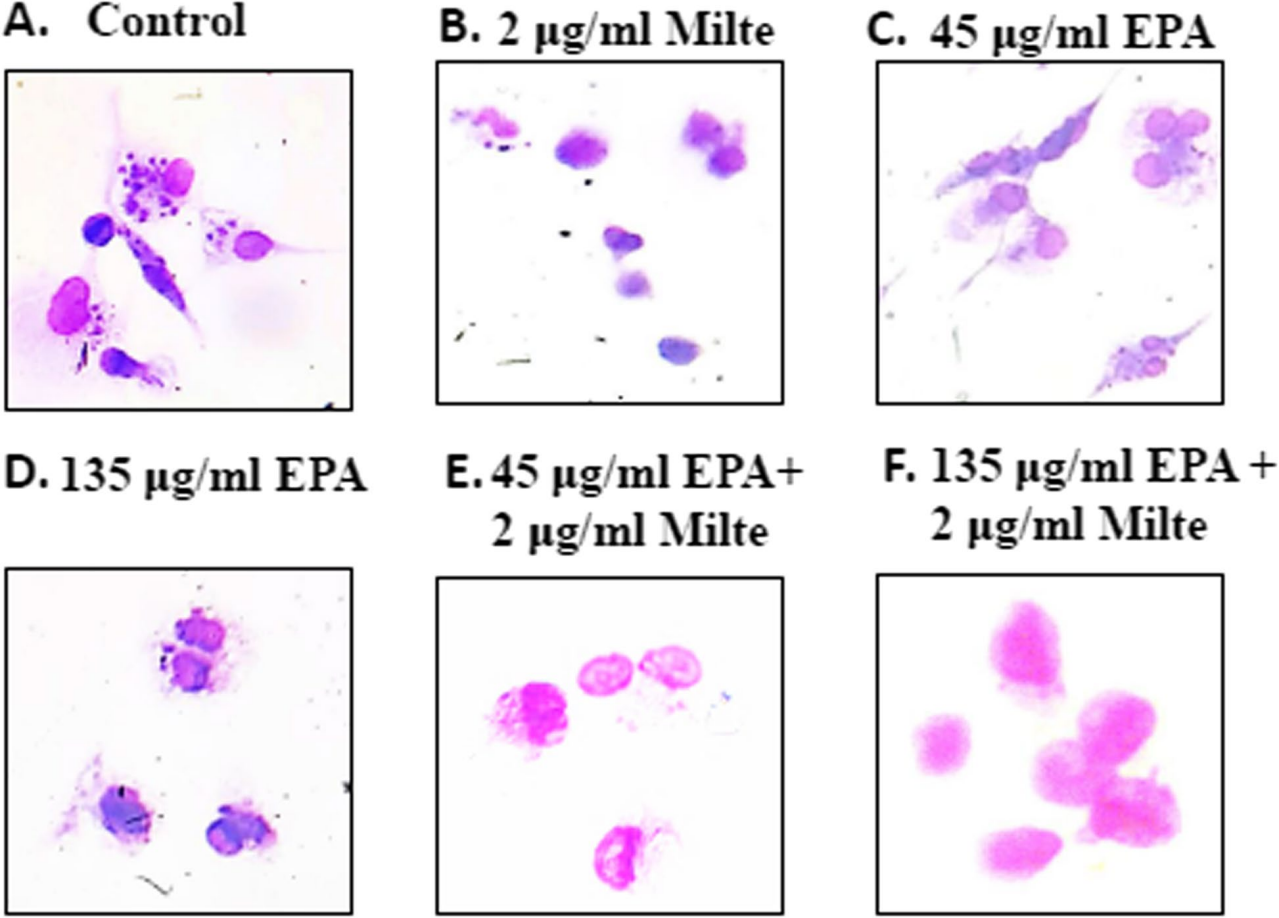
Photomicrographs illustrating images of *L.donovani* -infected macrophage stained with modified Giemsa stain. **A** Untreated control, **B** *Leishmania*-infected macrophages treated with 2μg/Ml of miltefosine, **C** *Leishmania*-infected macrophages treated with 45 μg/ml of EPA, **D** *Leishmania*-infected macrophages treated with 135 μg/mL of EPA, **E** *Leishmania*-infected macrophages treated with 45 μg/mL of EPA in combination with 2 μg/mL miltefosine, **F** *Leishmania*-infected macrophages treated with 135 μg/mL of EPA in combination with 2 μg/mL miltefosine.The images were captured at 100X under oil immersion

The promastigotes upon internalization are transformed into the amastigote form inside the parasitophorous vacuoles of macrophages. These amastigote forms of the parasites are non-motile and define the parasite pathogenicity. Thus, amastigotes being the biologically and clinically relevant form, it was indispensable to check the anti-amastigote efficacy of EPA. THP-1 differentiated macrophages were parasitized by *L. donovani* promastigotes and treated with different doses of the EPA alone and in combination with miltefosine. A significant reduction in the number of intracellular amastigotes was observed at 45 µg/ml, 135 µg/ml, and 250 µg/ml of EPA compared to untreated control (Fig. 3A). Further, findings reveal that the combinatorial doses of EPA with miltefosine significantly led to a reduction in the number of intracellular amastigotes compared to untreated control (Fig. 3B) The IC_50_ value on the intracellular amastigotes was found to be 49.65 ± 3.34 μg/ml and 19.54 ± 2.4 μg/ml for EPA treatment alone and with combination with miltefosine respectively (Fig. 3C&D). The Giemsa stained micrographs are representative images of antiamastigote assay of different doses of EPA alone and in combination with miltefosine (Fig. 4A-F).

### EPA alone as well as in combination with miltefosine augmented the expression of proapoptotic genes of *L. donovani*

**Fig. 5:**
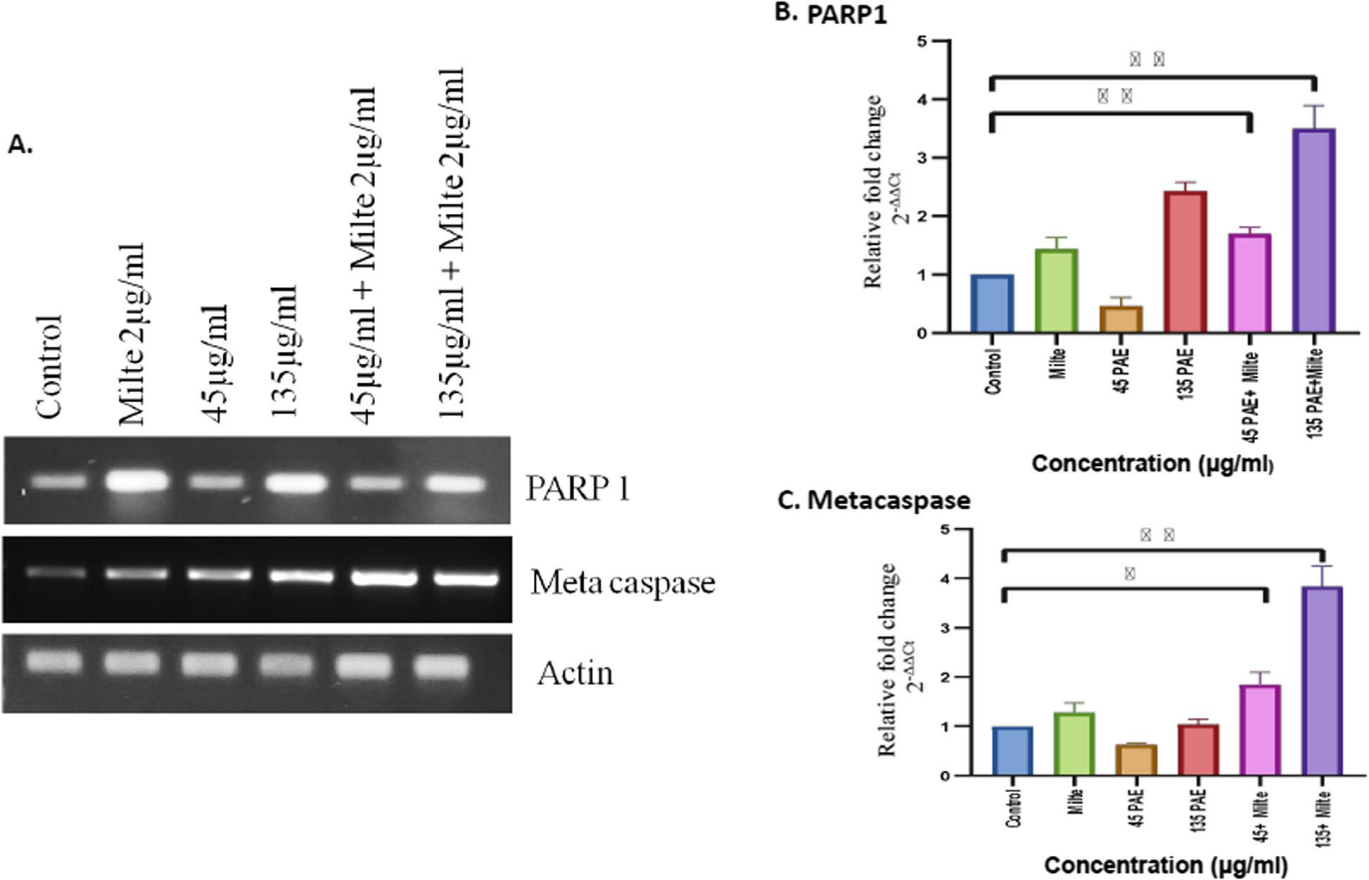
LdMetacaspase and LdPARP1 expression is up-regulated in *L.donovani on treatment with EPA *in combination with miltefosine. **A** Enhanced expression of LdPARP1 and LdMetacaspase was seen in parasites at combinatorial doses of miltefosine and EPA, as determined by RT-PCR gene expression study.** B** qRT-PCR-based analysis showed upregulated expression of PARP1 gene on treatment with increasing dose of EPA (45µg/ml to 135µg/ml) along with a constant dose of miltefosine (2 µg/ml) in *L. donovani.* **C **qRT-PCR revealed significant increased in relative mRNA expression levels of metacaspase in EPA + miltefosine treated *L.donovani* as compared to normal control. Results are presented as mean ± SEM

To further validate the role of EPA in the induction of apoptosis, apoptosis-related genes, LdMetacaspase and LdPARP1 were studied through RT-PCR and qRT-PCR. *L.donovani* promastigotes without treatment were taken as control whereas miltefosine alone was taken as positive control. The gene expression analysis through RT-PCR suggested enhanced expression of LdMetacaspase and LdPARP1 at combinatorial dosages of EPA and miltefosine in EPA-treated parasites (Fig. 5A; Suppl. Figure 1). Further, in qRT-PCR-based analysis, treatment of increasing dose of seed extract (45 µg/ml to 135 µg/ml) along with a constant dose of miltefosine (2 µg/ml) increased the expression of the proapoptotic gene of *L. donovani,* LdMetacaspase as compared to control. Similar results were obtained in the case of LdPARP1 gene (5B, C). These results corroborate that EPA combined with Miltefosine effectively inhibits *L.donovani* through apoptotic machinery.

### GC–MS analysis showed a variety of pharmacologically active phytoconstituents in EPA seed extract

Identification of plant secondary metabolites present in EPA was performed through GC–MS analysis. The total constituents found were 13 (Table 2), out of which the major constituents were Hydnocarpic acid (68.17%), Chaulmoogric acid (15.02%), Methyl 11-(2-cyclopenten-1-yl)undecanoate (7.72%), and Gorlic acid (3.25%). The peak areas of compounds represented the quantity proportions in the crude EPA (Suppl. Figure 2).

**Table 2:** Selectivity index of the extact

Parasite	Drug	CC50 (μg/ml)	IC50 (μg/ml)	SI INDEX
Promastigote	EPA	799.19	43.12	18.53
	EPA + Milte	384.16	4.547	84.48
Amastigote	EPA	799.19	49.65	16.09
	EPA + Milte	384.16	19.54	19.66

## Discussion

**Fig. 6:**
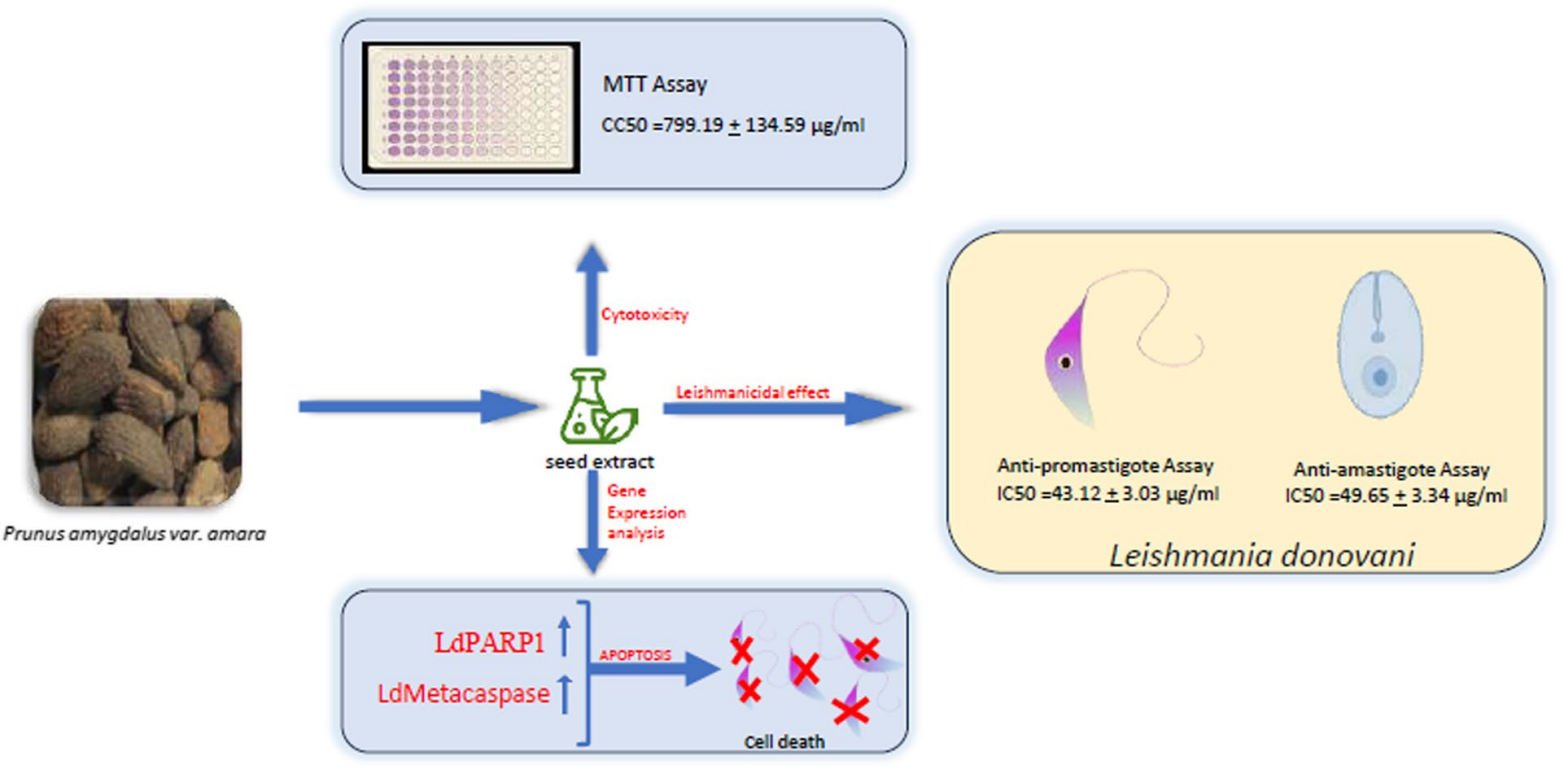
Diagrammatic representation of proposed mechanism of antileishmanial activity of EPA. EPA induced the apoptosis in the promastigotes through the upregulation of PARP-1 and metacaspase genes of *L. donovani*

The toxicity, resistance, and cost associated with the current antileishmanial drug regimen prompted us towards the exploration of ethnomedicines. Plant-based therapeutics have emerged as an alternative drug candidate for various diseases since lower cytotoxicity and higher efficacy are associated with it. *Prunus amygdalus var. amara* is one of the plants that holds substantial importance as a medicinal plant and the use of its root, seeds, and gum is described by Dioscorides [24]. A considerable number of flavonoids are present in the EPA, these flavonoids have been reported to have a role in the treatment of different types of cancer including colon, prostate, lung, breast, leukemia, and hepatocellular carcinoma [19]. However, the antileishmanial activity of EPA has not been explored yet. In this study, the mechanism of action of the antileishmanial activity of methanolic EPA was further investigated. Here, the in vitro anti-promastigote assay suggested a dose-dependent reduction in the number of *L. donovani* promastigotes, with an IC50 value of 43.12 + 3.03 μg/ml. Multiple reports suggested a combinatorial synergistic effect of EPA and plant-based compounds with standard drug miltefosine with lesser cytotoxicity [25, 26]. The synergistic antileishmanial effect of different Olive oil total polyphenolic fractions combined, with miltefosine has already been reported [27]. This study observed that a combinatorial dose of 45 μg/ml EPA with miltefosine significantly reduces the number of promastigotes with IC50 calculated to be 4.547 + 1.2 μg/ml. This finding suggests that EPA in combination with miltefosine is useful in the antileishmanial drug screening procedure. Although the plant has significant antileishmanial activity, the associatedpotential cytotoxicity should be addressed before proceeding with additional assays. Hence, an MTT test was conducted to assess the cytotoxicity of EPA alone and in combination with Miltefosine in THP-1 differentiated macrophages. It was found that the cytotoxicity levels were insignificant at concentrations where EPA has considerable anti-promastigote activity. This led to the conclusion that the concentration of 45 µg/ml EPA, 135 µg/ml EPA, 45 µg/ml EPA + Miltefosine, and 135 µg/ml EPA + Miltefosine can be used for further experiments. Since *Leishmania* is an intramacrophagic parasite that resides inside the macrophages and the amastigotes are in the infective stage it was pivotal to check the efficacy of the EPA against *Leishmania* amastigotes. A dose-dependent reduction in the number of intramacrophagic amastigotes. At 45 + miltefosine and 135 + miltefosine significant reduction was observed as compared to untreated control. Further, the expression of two *L.donovani*-specific pro-apoptotic genes LdMetacaspase and LdPARP1 were checked through RT-PCR and qRT-PCR. Dose-dependent increased expression of LdMetacaspase and LdPARP had already been reported in the liposomal formulation of HO-3867-treated inhibition of *Leishmania* growth [28]. Here, through RT-PCR and qRT-PCR it was observed that LdMetacaspase as well as LdPARP gene expression were significantly increased upon treatment with EPA + miltefosine. Together these results suggest and validate that EPA + miltefosine treatment significantly inhibited the growth of *Leishmania donovani* by the induction of apoptotic machinery.Almonds have long been recognized to contain a variety of bioactive substances. These substances might possess a range of antibacterial properties. Therefore, it was crucial to examine the different phytoconstituents of EPA. The GC–MS data have identified several phytochemicals with a variety of recognized anti-microbial characteristics. For example, it has been shown that chaulmoogric acid and hypocarpic acid limit the growth of mycobacteria [29, 30]. Additionally, a variety of fatty acids have been found. Furthermore, fatty acids have been found in prior studies as bioactive components that prevent the growth of mycobacteria, fungi, and malaria [31–33]. As a result, it can be strongly hypothesized that the phytochemical components separated from EPA might have potential antileishmanial properties (Fig. 6). Furthermore, more investigation can be carried out to clarify how these substances work against *Leishmania.*

## Conclusion

Plant extracts offer a promising avenue for treating leishmaniasis due to their antiparasitic properties, lower toxicity, potential to manage resistance, broader spectrum of activity, and cost-effectiveness. However, further research is needed to validate their efficacy, safety, and appropriate dosage for clinical use. Integrating plant-based therapies with conventional treatments may enhance overall efficacy and contribute to more sustainable and accessible solutions for combating leishmaniasis. Furthermore, based on the observed results, it can be concluded that the combination of EPA and miltefosine effectively inhibits *L. donovani* parasites by triggering apoptotic pathways. Moreover, EPA + miltefosine can be developed as an antileishmanial therapeutic after further validation in an in vivo system.

## Supplementary Information

Supplementary Material 1: Supplementary Fig. 1: Full gel images RT-PCR gene expression shown in Fig. 5A.
Supplementary Fig. 2: Chromatogram of GC–MS analysis of EPA.

## Data Availability

All the data and raw files are available with the first authors of the manuscript and will be provided upon the request.

## Abbreviations

CC50: 50% Cytotoxicity concentration
DMSO: Dimethyl sulfoxide
EPA: *Prunus**amygdalus** var. **amara* Seed extract
HEPES: 4-(2-Hydroxyethyl)-1-piperazineethanesulfonic acid
IC50: Half-maximal inhibitory concentration
SI: Selectivity index
MTT: 3-[4,5-Dimethylthiazol-2-yl]-2,5 diphenyl tetrazolium bromide
NTD: Neglected tropical disease
PBS: Phosphate-buffered saline
PMA: Phorbol 12-myristate 13-acetate
THP-1: Human acute monocytic leukemia cells
VL: Visceral leishmaniasis
