# Supplementary material for: Prunus amygdalus var. amara seed extract enhances the antileishmanial activity of miltefosine
Source: BMC Complement Med Ther. 2025 Jul 16;25:273. doi: 10.1186/s12906-025-04958-z (PMC12269125; doi:10.1186/s12906-025-04958-z)

Prunus amygdalus var. amara **seed extract enhances the antileishmanial activity of miltefosine**

Sajjadul Kadir Akand [a¶], Areeba Rahman[a¶], Rahat Ali[a], Mohammad Husain[a], Mohammad Danish[a] , Mohammad Rashid Khan[b], Nemat Ali[b], Mohd Faiz Akram[c], , Abdur Rub[a,*]

[a]*Department of Biotechnology, Jamia Millia Islamia (A Central University), New Delhi, India-110025.*

[b]*Department of Pharmacology and Toxicology, College of Pharmacy, King Saud University, P.O. Box 2457, Riyadh 11451, KSA*

[c]*Faculty of Dentistry, Department of Pharmacology, Jamia Millia Islamia (A Central University), New Delhi, India-110025,*

¶ Authors contributed equally

***Corresponding authors:**

Dr. Abdur Rub

Email I.D. arub@jmi.ac.in

Running title: Antileishmanial effect of bitter almond

Supp. Figure 1: Full gel image of RT-PCR gene expression shown in Figure 5 A.

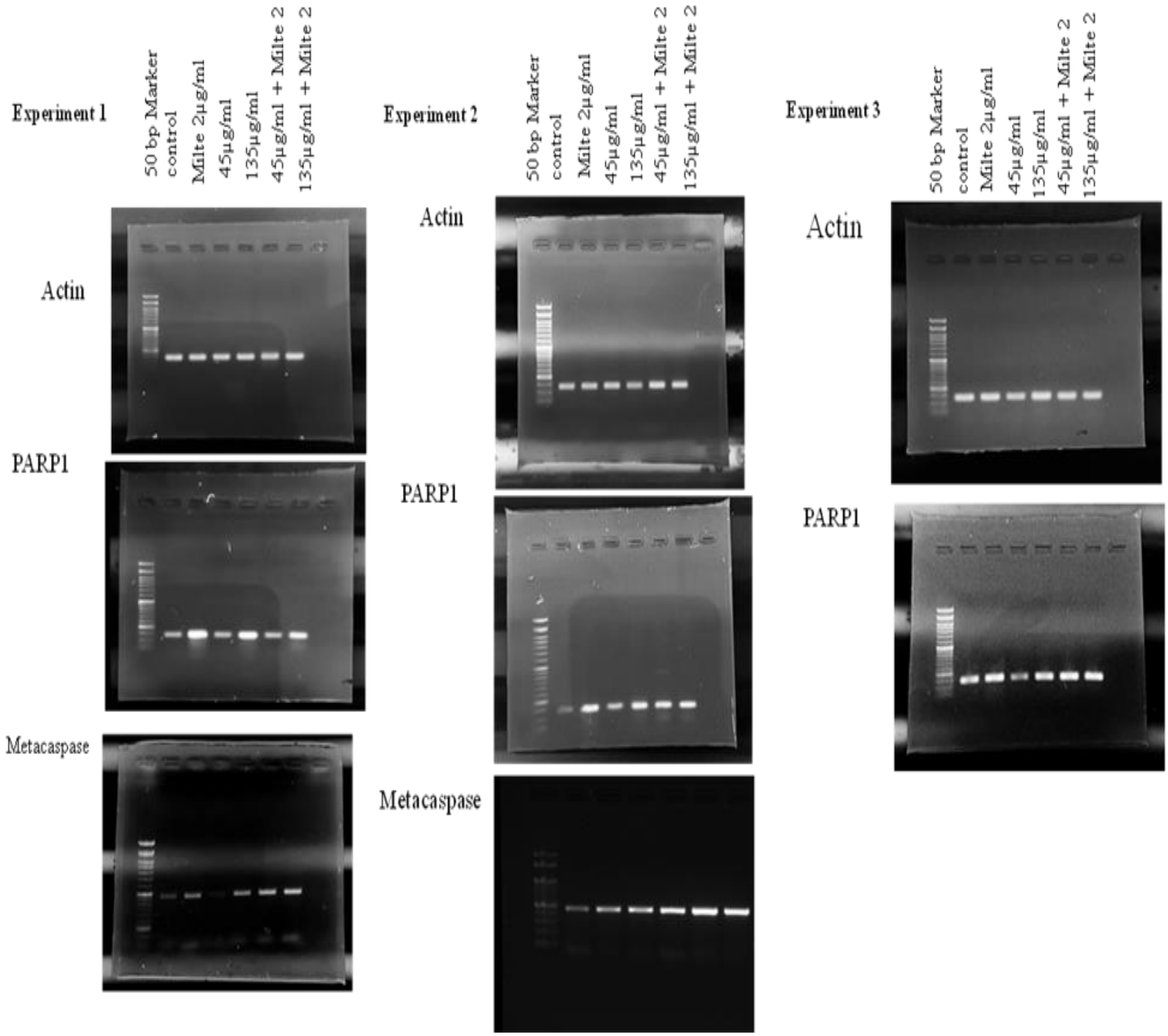

Supp. Figure 2: Chromatogram of GC-MS analysis of EPA.

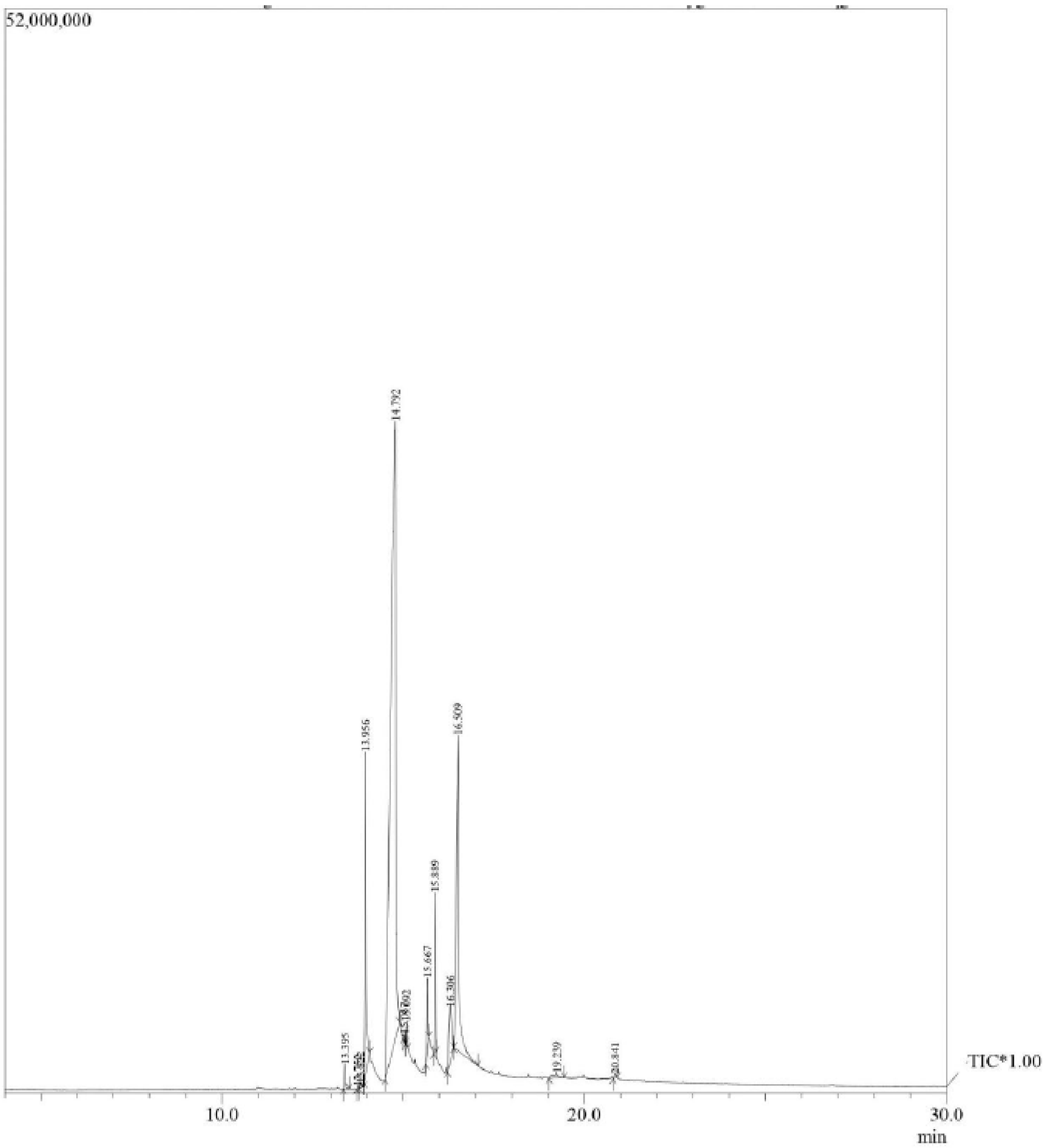

Supplement: Supplementary file 1 — Supplementary Material 1: Supplementary Fig. 1: Full gel images RT-PCR gene expression shown in Fig. 5A. Supplementary Fig. 2: Chromatogram of GC–MS analysis of EPA. [file 12906_2025_4958_MOESM1_ESM.pdf]
